# Higher Expression of CCL2, CCL4, CCL5, CCL21, and CXCL8 Chemokines in the Skin Associated with Parasite Density in Canine Visceral Leishmaniasis

**DOI:** 10.1371/journal.pntd.0001566

**Published:** 2012-04-10

**Authors:** Daniel Menezes-Souza, Renata Guerra-Sá, Cláudia Martins Carneiro, Juliana Vitoriano-Souza, Rodolfo Cordeiro Giunchetti, Andréa Teixeira-Carvalho, Denise Silveira-Lemos, Guilherme Corrêa Oliveira, Rodrigo Corrêa-Oliveira, Alexandre Barbosa Reis

**Affiliations:** 1 Laboratório de Imunopatologia, Núcleo de Pesquisas em Ciências Biológicas, Universidade Federal de Ouro Preto, Ouro Preto, Minas Gerais, Brasil; 2 Laboratório de Imunologia Celular e Molecular, Centro de Pesquisas René Rachou, Fundação Oswaldo Cruz, Belo Horizonte, Minas Gerais, Brasil; 3 Laboratório de Bioquímica e Biologia Molecular, Núcleo de Pesquisas em Ciências Biológicas, Universidade Federal de Ouro Preto, Ouro Preto, Minas Gerais, Brasil; 4 Departamento de Ciências Biológicas, Instituto de Ciências Exatas e Biológicas, Universidade Federal de Ouro Preto, Ouro Preto, Minas Gerais, Brasil; 5 Departamento de Análises Clínicas, Escola de Farmácia, Universidade Federal de Ouro Preto, Ouro Preto, Minas Gerais, Brasil; 6 Laboratório de Biomarcadores de Diagnóstico e Monitoração, Centro de Pesquisas René Rachou, Fundação Oswaldo Cruz, Belo Horizonte, Minas Gerais, Brasil; 7 Laboratório de Imunoparasitologia, Núcleo de Pesquisas em Ciências Biológicas, Universidade Federal de Ouro Preto, Ouro Preto, Minas Gerais, Brasil; 8 Laboratório de Parasitologia Celular e Molecular, Centro de Pesquisas René Rachou, Fundação Oswaldo Cruz, Belo Horizonte, Minas Gerais, Brasil; Institut Pasteur de Tunis, Tunisia

## Abstract

**Background:**

The immune response in the skin of dogs infected with *Leishmania infantum* is poorly understood, and limited studies have described the immunopathological profile with regard to distinct levels of tissue parasitism and the clinical progression of canine visceral leishmaniasis (CVL).

**Methodology/Principal Findings:**

A detailed analysis of inflammatory cells (neutrophils, eosinophils, mast cells, lymphocytes, and macrophages) as well as the expression of chemokines (CCL2, CCL4, CCL5, CCL13, CCL17, CCL21, CCL24, and CXCL8) was carried out in dermis skin samples from 35 dogs that were naturally infected with *L. infantum*. The analysis was based on real-time polymerase chain reaction (PCR) in the context of skin parasitism and the clinical status of CVL. We demonstrated increased inflammatory infiltrate composed mainly of mononuclear cells in the skin of animals with severe forms of CVL and high parasite density. Analysis of the inflammatory cell profile of the skin revealed an increase in the number of macrophages and reductions in lymphocytes, eosinophils, and mast cells that correlated with clinical progression of the disease. Additionally, enhanced parasite density was correlated with an increase in macrophages and decreases in eosinophils and mast cells. The chemokine mRNA expression demonstrated that enhanced parasite density was positively correlated with the expression of CCL2, CCL4, CCL5, CCL21, and CXCL8. In contrast, there was a negative correlation between parasite density and CCL24 expression.

**Conclusions/Significance:**

These findings represent an advance in the knowledge about skin inflammatory infiltrates in CVL and the systemic consequences. Additionally, the findings may contribute to the design of new and more efficient prophylactic tools and immunological therapies against CVL.

## Author Summary

Several previous studies correlated immunopathological aspects of canine visceral leishmaniasis (CVL) with tissue parasite load and/or the clinical status of the disease. Recently, different aspects of the immune response in *Leishmania*-infected dogs have been studied, particularly the profile of cytokines in distinct compartments. However, the role of chemokines in disease progression or parasite burdens of the visceralising species represents an important approach for understanding immunopathology in CVL. We found an increase in inflammatory infiltrate, which was mainly composed of mononuclear cells, in the skin of animals presenting severe forms of CVL and high parasite density. Our data also demonstrated that enhanced parasite density is positively correlated with the expression of CCL2, CCL4, CCL5, CCL21, and CXCL8. In contrast, there was a negative correlation between parasite density and CCL24 expression. These findings represent an advance in the knowledge of the involvement of skin inflammatory infiltrates in CVL and the systemic consequences and may contribute to developing a rational strategy for the design of new and more efficient prophylactic tools and immunological therapies against CVL.

## Introduction

Visceral leishmaniasis (VL), caused by *Leishmania* (*Leishmania*) *infantum* [syn. *Leishmania* (*Leishmania*) *chagasi*], is endemic in over 88 countries in Europe and Latin America and is transmitted by the bite of the female sand fly (phlebotomine) [1]. The skin is considered a key reservoir compartment for amastigotes in both asymptomatic and symptomatic *Leishmania*-infected dogs, and the important role of dogs in VL transmission in urban areas is supported by the high parasite loads found in the skin of infected animals and their shared habitat with humans [2]–[4]. Previous investigations have revealed that symptomatic *Leishmania*-infected dogs exhibit an intense diffuse dermal inflammatory infiltrate and a high parasitic burden in comparison with their asymptomatic counterparts [4]. On this basis it was proposed that the immunopathological changes in the skin and the levels of cutaneous parasitism are directly related to the clinical severity of the disease.

Several previous studies correlated immunopathological aspects of canine visceral leishmaniasis (CVL) with tissue parasite load and/or the clinical status of the disease [4]–[17]. The typical histopathological finding in tissues is a granulomatous inflammatory reaction associated with the presence of *Leishmania* amastigotes within macrophages [18]. In the skin of *Leishmania infantum*–infected dogs, the histopathological alterations consist of variable degrees of focal or diffuse inflammatory infiltrate in the dermis and variable numbers of plasma cells, macrophages, lymphocytes, and isolated neutrophils [19], [20].

Furthermore, it has recently been demonstrated that parasite density in the skin, bone marrow, and spleen compartments increases according to the severity of the clinical manifestation of CVL [6], [14], [16]. Calabrese et al. [21] evaluated histopathological aspects of the skin in naturally infected dogs and showed that low parasite load is associated with an intense inflammatory reaction driven mainly by mast cells, indicating that these cells exert a role in innate immunity and in the resistance against canine *Leishmania* infection.

Recently, different aspects of the immune response in *Leishmania*-infected dogs have been studied, particularly the profile of cytokines in distinct compartments [5], [9], [12], [17], [22]–[24]. However, the role of chemokines in disease progression or parasite burdens of the visceralising species represents an important approach for understanding immunopathology in CVL.

Chemokines are chemotactic factors that coordinate recruitment of leukocytes that are involved in homeostasis as well as innate and adaptive immune responses. In the context of experimental or natural infection in CVL, an up-regulation of the chemokines in the spleen has been described, although only CXCL10 and CCL5 were markedly elevated in oligosymptomatic dogs [24]. In addition, the augmented levels of chemokines suggested an accumulation of infiltrating monocytes attracted by CCL3 and CCL2. CD4^+^Th1 and CD8^+^ cells also accumulated and may have been recruited by CXCL10, with further expression induced through IFN-γ secretion [24].

Considering the importance of chemokines on the pattern of CVL and the lack of studies on this topic, understanding the chemokine profile during ongoing *L. infantum* infection in dogs is a prerequisite for identifying the mechanisms for resistance or susceptibility in this experimental model. In the present study, the immunopathology of CVL was investigated by performing detailed analyses of the RNA expression of different chemokines (CCL2, CCL4, CCL5, CCL13, CCL17, CCL21, CCL24, and CXCL8) and the occurrence of inflammatory cells (neutrophils, eosinophils, mast cells, lymphocytes, and macrophages). We focused on selected chemokines in order to characterize their role in recruiting particular cell types to the inflammatory infiltrate in skin from *L. infantum*–infected dogs. Thus, we found that the chemokines CCL2, CCL4, CCL5, and CCL21 attract macrophages; CCL5 and CCL4 attract inflammatory lymphocytes, particularly Th1-type cells; CCL24 attracts eosinophils; and CXCL8 attracts neutrophils, monocytes, and lymphocytes. The chemokine CCL17 helps to establish the inflammatory infiltrate, a characteristic feature of various inflammatory skin conditions, by attracting CCR4-bearing cells, which are especially polarized to Th2-type cells and regulatory T cells [25]. This study was carried out using the skin from 35 dogs that were naturally infected with *L. infantum*.

## Materials and Methods

### Study population and clinical evaluation

The study was approved by the Committees of Ethics in Animal Experimentation of the Universidade Federal de Ouro Preto (protocol no. 083/2007) and of the Universidade Federal de Minas Gerais (protocol no. 020/2007) and the City Council of Belo Horizonte (protocol no. 001/2008). All procedures in this study were according to the guidelines set by the Brazilian Animal Experimental Collage (COBEA), Federal Law number 11794. The study population comprised 51 adult dogs (aged between 2 and 6 years) of both sexes that had been captured by the Center of Zoonosis Control in Belo Horizonte (Minas Gerais, Brazil), a region with a high prevalence of CVL and human VL. The animals were maintained under quarantine at the kennels of the Instituto de Ciências Biológicas (Universidade Federal de Minas Gerais) prior to tissue collection for 40 days and treated for intestinal helminthic infections (Endal Plus; Schering-Plough Coopers, São Paulo, SP, Brazil). We treated kennels with pyrethroid insecticide monthly during the quarantine and throughout the experiments. Experimental animals were categorized on the basis of serological results from an indirect immunofluorescence assay test, the “gold standard” immunological test in Brazil for the diagnosis of CVL. Sixteen dogs with negative immunofluorescence assay test results from serum samples diluted 1∶40 and negative results for *Leishmania* in tissue smears (bone marrow, ear skin, spleen, liver, and popliteal lymph node) were considered to be non-infected and were used as the control group (CD, *n* = 16). Thirty-five animals with positive immunofluorescence assay titers ≥1∶40 were considered CVL positive and comprised the infected animal groups. The infected animal groups were subdivided on the basis of the presence or absence of signs of infection according to Mancianti et al. [25] as follows: an asymptomatic group (AD, *n* = 10), in which indicative signs of the disease were absent; an oligosymptomatic group (OD, *n* = 10), in which a maximum of three clinical signs of the disease were present, including opaque bristles, localized alopecia, and/or moderate weight loss; and a symptomatic group (SD, *n* = 15), in which characteristic clinical signs of the disease were present, including cutaneous lesions, onycogryphosis, opaque bristles, severe loss of weight, apathy, and keratoconjunctivitis.

### Sample collection and assessment of skin parasite load

Animals were euthanized with sodium thiopental (Abbott Laboratories, Abbott Park, IL, USA; 30 mg/kg body weight) and samples of skin tissue were collected from ear areas without lesions. A skin fragment from each group was used for tissue imprints on microscopic slides coded for blinded analysis. The samples were fixed in methanol, stained with Giemsa, and examined under an optical microscope. *Leishmania* amastigote stages were counted and parasite densities were expressed as *Leishman* Donovan Units (LDU), corresponding to the number of *Leishmania* amastigotes per 1000 nucleated cells per skin imprint as described by Stauber [27], with some modifications according to Reis et al. [7], [8]. Parasite densities were categorized statistically into tertiles as absent (LDU = 0; CD group, *n* = 16), low (LDU = 1–9; LP group, *n* = 12), medium (LDU = 10–130; MP group, *n* = 11), and high (LDU = 131–7246; HP group, *n* = 12).

### Extraction of total RNA and synthesis of first strand cDNAs

A second fragment of ear skin was stored at −80°C until required for RNA analysis. Total RNA was extracted by homogenizing approximately 20 mg of skin tissue with 1 mL of TRIzol reagent (Invitrogen Brasil, São Paulo, SP, Brazil) in a rotor stator. The lysate was incubated at room temperature for 10 min, mixed with chloroform (200 µL) by tube inversion, and centrifuged at 12,000× *g* for 10 min at 4°C. The aqueous phase was collected, and RNA extraction was done by using the SV Total RNA Isolation System (Promega, Madison, WI, USA) according to the manufacturer's recommendations, which included a DNase treatment step. The efficiency of DNAse treatment was evaluated by PCR amplification of the cDNA reaction mix without the addition of the ThermoScript enzyme. Finally, each quantitative PCR (q-PCR) run was performed with two internal controls assessing both potential genomic DNA contamination (no reverse transcriptase added) and purity of the reagents used (no cDNA added). Strand cDNAs were synthesized from 1.0 µg of total RNA using the ThermoScript RT-PCR System (Invitrogen Brasil) with oligo-dT primers according to the manufacturer's instructions.

### Design of primers for gene evaluation

Primers were designed with the aid of Gene Runner version 3.05 (Hasting Software Inc. 2004) using specific canine sequences obtained from GenBank (http://www.ncbi.nlm.nih.gov/genbank/). The sequences of the primers are listed in Table 1. The primers were synthesized by Eurogentec (Southampton, UK) and reconstituted in nuclease-free water.

**Table 1:** Sequences of primers used for quantification of mRNA expression by real-time PCR^a^.

Gene	Primer sequence (5′–3′)	Product length (bp)	GenBank accession no.	Reaction efficiency (%)	*R* ^2^
GAPDH		115	AB038240	99.1	0.996
F	TTCCACGGCACAGTCAAG				
R	ACTCAGCACCAGCATCAC				
CCL2		91	U29653	96.3	0.979
F	CCTGCTGCTATACACTCA				
R	GCTTCTTTGGGACACTTG				
CCL4		76	AB183194	95.9	0.981
F	TCCTACTGCCTGCTGCTT				
R	GCTGGTCTCAAAGTAATCTGC				
CCL5		136	AB098562	98.7	0.991
F	TTCTACACCAGCAGCAAG				
R	TTCTACACCAGCAGCAAG				
CCL13		84	AB162849	97.6	0.987
F	CCCTATTCACTTGCTGCTT				
R	AGTGGCTGCTGGTGATTC				
CCL17		128	AB054642	95.4	0.967
F	TCCAAGGCAAGTCCATCT				
R	GAGGTCTCCAAATGATCCA				
CCL21		60	AB164433	96.7	0.972
F	AGTCTGGCAAGAAGGGAAAG				
R	GGGTCTGTGGCTGTTCAGT				
CCL24		149	AB162851.1	95.3	0.968
F	CCTAAGGCAGGAGTGGTCTT				
R	AGGGCTTTGGTGCTCATTG				
CXCL8		116	AF048717	98.7	0.977
F	ACACTCCACACCTTTCCAT				
R	GGCACACCTCATTTCCATTG				

a F: Forward primer, R: Reverse primer. GenBank accession number of the sequence used to design primers and their product length are shown, as well as each PCR efficiency and *R^2^*.

### Real-time PCR and cloning and sequencing of amplicons

q-PCR was performed on an ABI Prism 7000 DNA Sequence Detection System using SYBR Green PCR Master Mix (PE Applied Biosystems, Foster City, CA, USA), with 100 mM of each primer and cDNA diluted to 1∶5. The samples were incubated at 95°C for 10 min and then submitted to 40 cycles of 95°C for 15 s and 60°C for 1 min, during which time fluorescence data were collected. The efficiency of each pair of primers was evaluated by serial dilution of cDNA according to the protocol developed by PE Applied Biosystems. In order to evaluate gene expression of the chemokines CCL2, CCL4, CCL5, CCL13, CCL17, CCL21, CCL24, and CXCL8, three replicate analyses were performed, and the amount of target RNA was normalized with respect to the endogenous control (housekeeping) gene GAPDH. Data were expressed according to the 2^−ΔΔCt^ method using the mean value of the ΔCt of the control group as the calibrator [26]. After normalization, the expression levels of chemokines in the infected groups were considered up-regulated or down-regulated compared to expression levels in the control group. PCR products were cloned with pGEM-T Easy Vector (Promega) and sequenced to check specificity by using an ABI 3100 Automated Sequencer (PE Applied Biosystems) and a Dye Terminator Kit.

Table 2 presents a summary of the different chemokines and their biological effects during *Leishmania* infection in dogs, mice, and humans. These data illustrate how recruitment of specific cells might influence the pathogenesis of *Leishmania* infection.

**Table 2:** Chemokines in *Leishmania spp*. infection.

Model infection	Chemokine	*Leishmania* species	Biological effect	Ref.
**Canine infection**				
Naturally infected	CCL5CXCL10	*L. infantum*	Increase levels in the spleen and associated a high IFN-γ expression	[24]
Experimentally infected(*in vivo*)	CCL2CCL3CXCL10	*L. infantum*	Suggest that occurs increased recruitment of infiltrating monocytes attracted by CCL2 and CCL3, as well as of CD4^+^T_H_1 and CD8^+^ cells which could have been recruited by CXCL10 and induced further CXCL10 expression through IFN-γ secretion	[24]
**Human and murine infection**				
Naturally infected	CCL7CCL17	*L. braziliensis*	Higher expression of CCL7 and CCL17 in lesions from late localized cutaneous leishmaniasis (CL) and higher frequency of CCL7 in difuse CL lesions suggesting a preferential recruitment of regulatory T cells in the late phase of the infection	[52]
	EotaxinCXCL8	*L. donovani*	Increase of eosinophil and neutrophil turnover and activity in patients with VL relate to the reduced production and availability of the chemokines Eotaxin and CXCL8	[54]
Experimentally infected(*in vitro*)	CCL2	*L. infantum*	Before stimulated human macrophages experimentally infected with *L. infantum* with CCL2, levels of nitric oxide produced were similar to those obtained by stimulation with IFN-γ, which increased the ability of these cells to eliminate the parasite	[34]
	CCL2, 3	*L. infatum* *L. donovani*	Induce leishmanicidal ability in vitro in human macrophages infected by *L. infantum* and can control the growth and multiplication of intracellular *L. donovani* via regulatory mechanisms mediated by nitric oxide	[35]
Experimentally infected(*in vivo*)	CCL3CCL4CCL5	*L. amazonensis*	Reductions in expression of these mediators accompanied by reduced T-cell response in susceptible mice	[33]
	CCL19, 21	*L. donovani*	In murine infection, CCL21 is important in the marginal zone of the spleen for maintaining this cellular structure and capturing the blood antigens and greater susceptibility to infection due to the loss of dendritic cell migration	[37]–[38]
	CCL2	*L. donovani*	In initial phase of infection, attract monocytic cells	[53]
	CXCL10	*L. donovani*	Attract lymphocytes and associated to granuloma formation in liver	[53]
	CCL2	*L. infantum*	Recruitment of monocytes in the spleen and sustained parasite persistence	[55]

### Histological analysis

For standard histological examination (morphometric analysis and leukocyte differential counting) sections were coded and stained with hematoxylin and eosin and subsequently underwent blinded analysis under an optical microscope (model CH3RF100, Olympus Optical Co., Tokyo, Japan). The inflammatory cells (neutrophils, eosinophils, macrophages, mast cells, and lymphocytes) that were recruited to the dermis were counted, and the results are expressed in percentages. Cell types in the cellular infiltrate in the dermis were quantified by using 20 random images (total area = 1.5×10^6^ µm^2^) that adequately represented a slide. Thus, the density and predominance of cells in the inflammatory infiltrate and their distribution within the skin layers were assessed and registered quantitatively. The images displayed in the 40× objective were digitized through a Leica DM5000B microscope with a coupled camera using the program Leica Application Suite (version 2.4.0 R1, Leica Microsystems Ltd., Heerbrugg, Switzerland). For the analysis of images, Leica QWin V3 (Leica Microsystems Ltd.) was used to count all cell nuclei, excluding the pilose follicles, skin annexes, and epidermal cells.

### Statistical analysis

Statistical analyses were performed using the GraphPad Prism software package version 5.0 (GraphPad Software, San Diego, CA, USA). Normality of the data was established using the Kolmogorov-Smirnoff test. The Kruskal-Wallis test was used for comparative studies between groups, followed by Dunn's test for multiple comparisons. Spearman's rank correlation was also computed in order to investigate relationships between the expression of chemokine mRNAs with clinical forms and skin parasite density as well as cell counts. In all cases, differences were considered significant when the probabilities of equality (*p* values) were ≤0.05.

## Results

### Clinical progression in CVL was correlated with increased parasite density and the presence of mononuclear cells in the skin of dogs naturally infected by *L. infantum*

**Figure 1:**
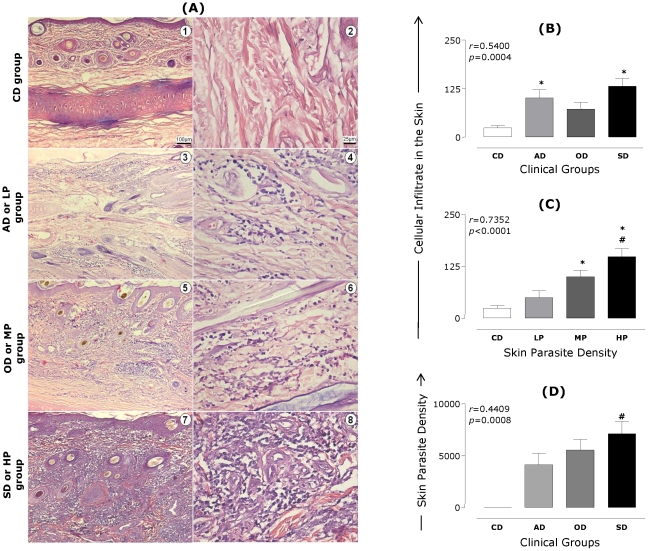
Histopathological and parasite density analyses of skin of dogs naturally infected with *L. infantum*. Animals were categorized according to their clinical status into asymptomatic (AD, *n* = 10), oligosymptomatic (OD, *n* = 10), or symptomatic (SD, *n* = 15) or categorized according to skin parasite density into low (LP, *n* = 12), medium (MP, *n* = 11), or high (HP, *n* = 12) parasite density. The control group is represented by CD (*n* = 16). Photomicrographs of cutaneous cellular infiltrates from dogs naturally inflected by *L. infantum* stained by hematoxylin and eosin (**A**). Representative cellular infiltrates of study groups are depicted: Control dogs (1 and 2 ); AD or LP (3 and 4); OD or MP (5 and 6); SD or HP (7 and 8). **Left panels:** Slides shown at 10× magnification; bar, 100 µm. **Right panels:** Slides shown at 40× magnification; bar, 25 µm. Correlation between quantitative analysis of cutaneous cellular infiltrate with clinical status (**B**) or skin parasite density (**C**) are presented. Correlation between clinical groups and skin parasite density is also presented (**D**). The results are expressed as the mean number of cells present in cutaneous cellular infiltrates evaluated at 20 fields plus standard deviation. In (**D**), the results are expressed as the mean of the log number of skin parasite density plus standard deviation. Significant differences (*p*<0.05) compared with CD and AD or LP groups are indicated by symbols * and #, respectively. Spearman's correlation indexes (*r* and *p* values) are shown on the graphs when applicable.

In order to investigate the relationship between clinical forms of CVL and skin parasite density as well as cellular infiltrate, correlation analyses were conducted with these parameters in *L. infantum*–infected animals (*n* = 35) (Fig. 1). The main histopathological findings are shown in photomicrographs (Fig. 1A). Histopathological examination of the skin showed no histological changes within the CD group (Fig. 1A, panels 1 and 2). In the LP and AD groups (Fig. 1A, panels 3 and 4), there was a mild inflammatory infiltrate, composed mainly of mononuclear cells, while in the OD and MP groups, this infiltration was mild to moderate, as shown in Fig. 1A (panels 5 and 6). In panels 7 and 8 of Fig. 1A, which represent sections of ear skin in the SD group, an intense cellular infiltrate composed mainly of mononuclear cells was observed.

The intensity and predominance of cells in the inflammatory infiltrate and their distribution within the skin layers were assessed (Fig. 1B, 1C). Our results demonstrated a positive correlation between cellular infiltrate and clinical status (*r* = 0.5400, *p* = 0.0004) (Fig. 1B) and skin parasite density (*r* = 0.7352, *p*<0.0001) (Fig. 1C). Significant increases in the inflammatory infiltrate in the skin samples were observed in the AD and SD groups as compared with CD animals (Fig. 1B). The HP group had a significant increase in inflammatory infiltrate compared with the CD and LP groups (Fig. 1C). Moreover, the inflammatory infiltrate within the MP group was significantly increased as compared with the CD group (Fig. 1C).

The results also indicated positive correlation among clinical evolution of CVL and the increase of parasite density in the skin (*r* = 0.4409, *p* = 0.0080) (Fig. 1D). Additionally, an increase in parasite density (*p*<0.05) was detected in the skin of dogs showing the maximum clinical score (SD) when compared with the AD group (Fig. 1D).

### Assessment of the inflammatory cell profile in the skin revealed an increase in macrophages and reductions in lymphocytes, eosinophils, and mast cells according to the clinical progression of CVL

**Figure 2:**
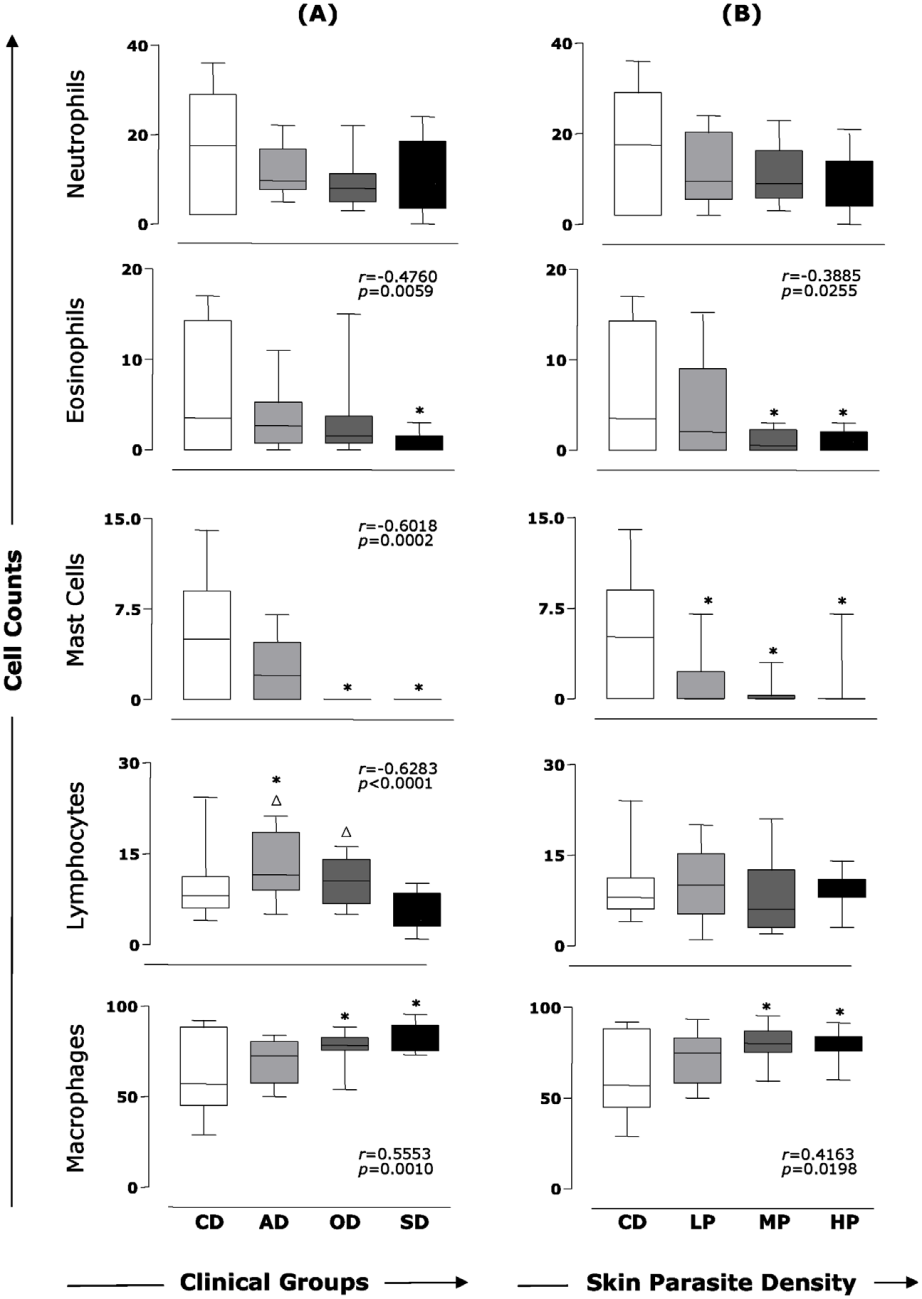
Cellular profile of the skin from dogs naturally infected by *L. infantum*. (**A**) Animals were categorized according to their clinical status into asymptomatic (AD, *n* = 10), oligosymptomatic (OD, *n* = 10), or symptomatic (SD, *n* = 15) or categorized according to skin parasite density into low (LP, *n* = 12), medium (MP, *n* = 11), and high (HP, *n* = 12) parasite density. The control groups are represented by CD (*n* = 16). Relationship between clinical forms and percentage of cells in the skin of dogs naturally infected with *L. infantum* (**A**). Relationship between skin parasite density and percentage of cells in the skin of dogs naturally infected with *L. infantum* (**B**). The data are presented as boxplots. The box stretches from the lower hinge (defined as the 25th percentile) to the upper hinge (the 75th percentile) and therefore contains the middle half of the score in the distribution. The median is shown as a line across the box. Therefore, one fourth of the distribution is between this line and the bottom or the top of the box. Significant differences (*p*<0.05) compared with CD and SD or HP are indicated by symbols * and *Δ*, respectively. Spearman's correlation indexes (*r* and *p* values) are shown on the graphs when applicable.

The study of skin tissue cellularity included an assessment of the percentage of cell types (neutrophils, eosinophils, mast cells, lymphocytes, and macrophages) present in the inflammatory infiltrate in the skin of dogs that were naturally infected by *L. infantum* and categorized by clinical status and dogs that were uninfected (Fig. 2A). In this context, we observed a reduction in the percentage of eosinophils in the SD group compared with the CD group (*p*<0.05), and a negative correlation between this cell population (*r* = −0.4760, *p* = 0.0059) and clinical status. Additionally, there was a decrease (*p*<0.05) in the percentage of mast cells in the OD and SD groups when compared with the CD group. Similarly, a negative correlation was observed in the percentage of mast cells (*r* = −0.6018, *p* = 0.0002) compared with the clinical form of CVL. For lymphocytes, we observed an increased (*p*<0.05) percentage in the AD group in comparison with the SD group and control dogs. Furthermore, we also observed an increase (*p*<0.05) in the OD group as compared with the SD group. The analysis of correlation between lymphocyte counts and clinical status showed a negative correlation between the increase of lymphocytes versus the clinical outcome in CVL (*r* = −0.6283, *p*<0.0001) (Fig. 2A). Significant increases (*p*<0.05) were observed in the OD and SD groups in the population of macrophages in comparison to the CD group, and a positive correlation was observed (*r* = 0.5553, *p*<0.0010) between the percentage of macrophages and degree of disease.

### Enhanced skin parasite density was correlated with an increase of macrophages and decreases of eosinophils and mast cells in the skin of dogs naturally infected by *L. infantum*

An assessment of cellular infiltrate in the skin of dogs naturally infected by *L. infantum* and uninfected dogs was performed by categorizing them according to skin parasite density (Fig. 2B). Although neutrophil and lymphocyte subsets did not have significant changes, a shift in the cell profiles related to the innate immune response was observed. The percentage of eosinophils decreased (*p*<0.05) in the MP and HP groups when compared with the CD group. Associated with these observations, a negative correlation between the percentage of eosinophils and skin parasite density was found (*r* = −0.3885, *p* = 0.0255). The percentage of mast cells was lower in the LP, MP, and HP groups when compared with the CD group (*p*<0.05). Accordingly, a significant increase (*p*<0.05) in the percentage of macrophages in the MP and HP groups in comparison with the CD group was found. Furthermore, a positive correlation between the percentage of macrophages and skin parasite density (*r* = 0.4163, *p* = 0.0198) was observed.

**Figure 3:**
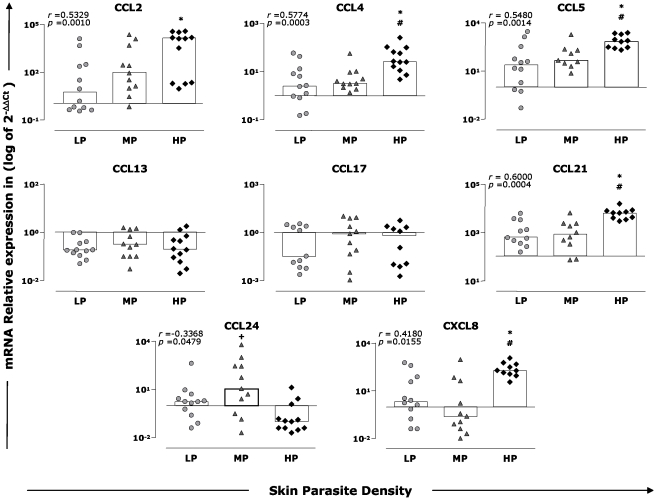
Skin parasite density and expression of chemokines in the skin from dogs with *L. infantum*. Animals were categorized according to their skin parasite density into low (LP = gray circles), medium (MP = gray triangles), and high (HP = dark diamonds) parasite load. The results are expressed as scattering of individual values and median of that group (bars) of log_10_ of the relative copy number of mRNA for CCL2, CCL4, CCL5, CCL13, CCL17, CCL21, CCL24, and CXCL8. Significant differences (*p*<0.05) compared with LP, MP, and HP are indicated by the symbols *, #, and +, respectively. Spearman's correlation indexes (*r* and *p* values) are shown on the graphs when applicable.

Enhanced parasite density was positively correlated with higher expression of chemokines CCL2, CCL4, CCL5, CCL21, and CXCL8 and lesser expression of CCL24 in the skin of dogs naturally infected by *L. infantum*. The involvement of chemokines in recruiting cells to the skin and developing a protective response against *Leishmania* infection was evaluated according to skin parasitism. These results are described in Figure 3. In this study, we also performed correlation analysis between the levels of chemokine expression and the clinical status, but significant differences did not exist between the groups (data not shown). The mRNA expression of CCL2 was increased (5.8-fold; *p*<0.05) in the HP group as compared with the LP group. Furthermore, the correlation analysis showed that CCL2 was positively associated with an increase of parasite load in the skin of these animals (*r* = 0.5329, *p* = 0.0010). CCL4 was up-regulated in all groups in relation to the CD group and highly expressed in the HP group in comparison to the LP and MP groups (3.5-fold and 2.8-fold, respectively; *p*<0.05). Additionally, a positive correlation (*r* = 0.5774, *p* = 0.0003) with an increase in skin parasite density was detected. Similarly, CCL5 expression indicated a significant up-regulation occurred in all infected groups when compared to CD, and increased levels were observed in the HP group in comparison with the LP and MP groups (2.1-fold and 1.7-fold, respectively; *p*<0.05). Moreover, a positive correlation could be established between the expression of CCL5 and skin parasite density (*r* = 0.5480, *p* = 0.0014).

CCL13 and CCL17 were down-regulated in all groups in comparison to the CD group; however, significant differences were not found between experimental groups. For the chemokine CCL21, we observed increased expression in all groups compared with the CD group, and high levels were found in the HP group compared with the LP and MP groups (1.4-fold and 1.3-fold, respectively; *p*<0.05). In addition, a positive correlation was observed between CCL21 expression and parasite density (*r* = 0.6000, *p* = 0.0004).

The expression of CCL24 was up-regulated in the LP and MP groups and down-regulated in the HP group compared to the CD group. In addition, higher CCL24 expression was observed in the MP group when compared with the HP group (0.9-fold; *p*<0.05). Furthermore, a negative correlation was observed between CCL24 expression and skin parasite density (*r* = −0.3368, *p* = 0.0479). With regard to CXCL8, we observed an increase in the target transcript levels in the LP and HP groups and down-regulation in the MP group compared with CD. In addition, CXCL8 expression was significantly higher in the HP group compared with the LP and MP groups (6.9-fold and 8.3-fold, respectively; *p*<0.05). Positive correlation was observed between CXCL8 expression and skin parasite density (*r* = 0.4180, *p* = 0.0155).

### Skin parasite density was most strongly correlated with chemokines that induce macrophage migration during CVL

**Figure 4:**
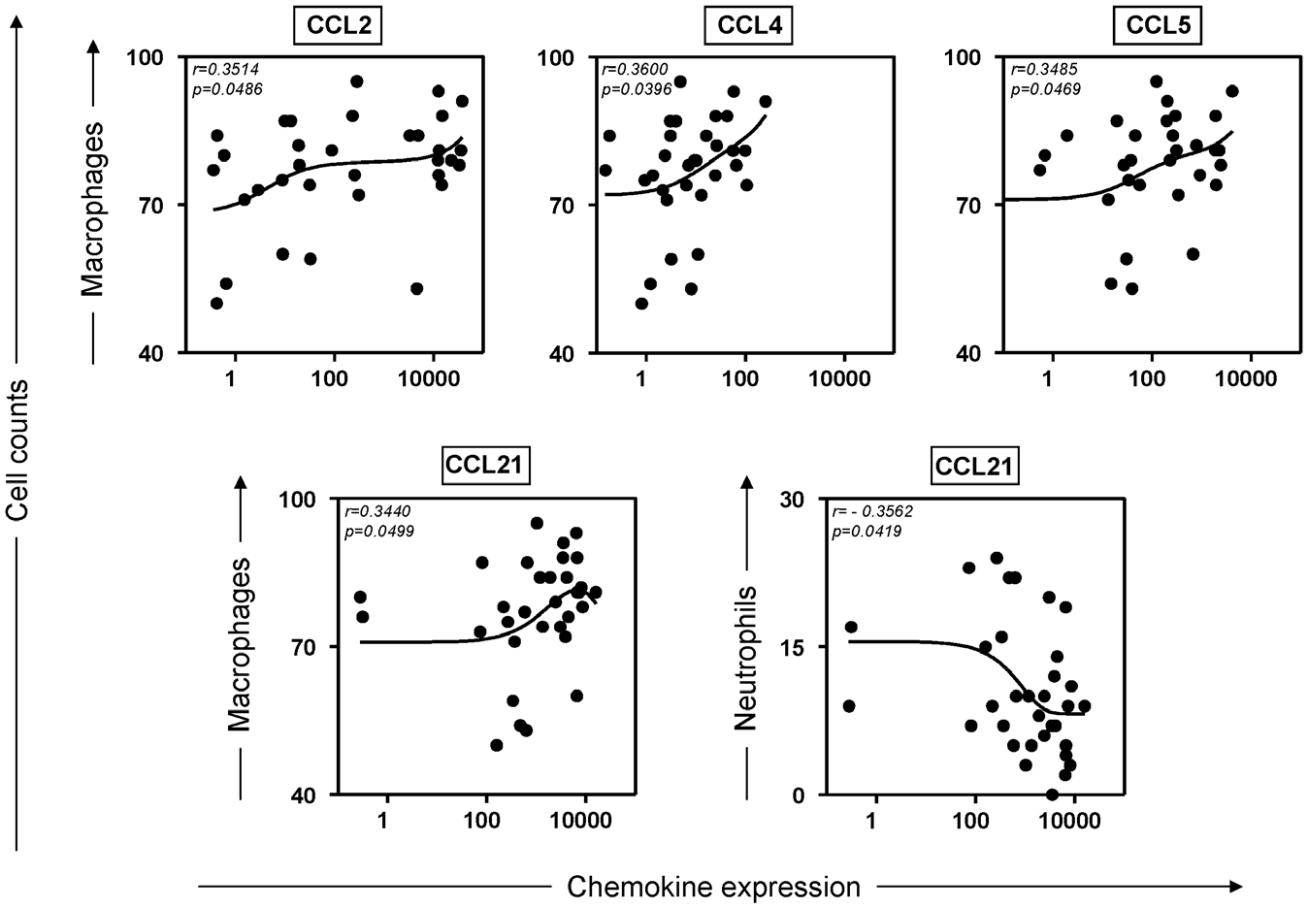
Correlation between parasite density (LDU) and cell-types presenting into skin from *L. infantum* infected dogs. The results were expressed on graphs as scattering of individual values. Spearman correlation indexes (*r*) at *p*<0.05 are shown on graphs. Connecting lines illustrated positive and negative correlation indexes.

In order to better identify the association between inflammatory cells present in skin and chemokine levels, we performed additional correlation analyses between distinct cell types and cutaneous chemokine expression (Fig. 4). Interestingly, our results indicated that macrophages were the cell type that was most likely to be recruited by chemokines CCL2 (*r* = 0.3514; *p* = 0.0486), CCL4 (*r* = 0.3600; *p* = 0.0396), CCL5 (*r* = 0.3485; *p* = 0.0469), and CCL21 (*r* = 0.3440; *p* = 0.0499) (Fig. 4). Negative correlation was observed between CCL21 levels and neutrophils (*r* = −0.3562; *p* = 0.0419).

## Discussion

The analysis of chemokine expression in lymphoid compartments is crucial for assessing central regulation and pathophysiological processes, including traffic homeostasis, inflammation, and hematopoiesis [27], [28]. In this context, few studies have investigated the levels of chemokines in ongoing CVL. In one of these studies, Strauss-Ayali et al. [24] evaluated the expression of CCL2, CCL4, CCL5, and CXCL10 in the spleen of dogs naturally or experimentally infected by *L. infantum* and found an increase of CCL2 and CCL5 in the experimentally infected dogs.

Herein, increased levels of CCL2, CCL4, and CCL5 in dogs with high parasitism were observed, and these chemokines were positively correlated with parasite density. These results indicate a preferential migration of macrophages into the skin, suggesting a host strategy to control parasitism during ongoing CVL. It has been proposed that in leishmaniasis, chemokines CCL2, CCL4, and CCL5 generally play a role not only as chemotactic factors but also as co-activators of macrophages and consequently have a part in the elimination of parasites [29]–[32].

After stimulation with CCL2, human macrophages experimentally infected with *L. infantum* produced levels of nitric oxide that were similar to those obtained by stimulation with IFN-γ, which increased the ability of these cells to eliminate the parasite [33]. In addition, CCL2 and CCL3 may induce leishmanicidal ability *in vitro* in human macrophages infected by *L. infantum* and can control the growth and multiplication of intracellular *L. donovani* via regulatory mechanisms mediated by nitric oxide [34]. In the present work, we demonstrated an increase in the percentage of macrophages in dogs with clinical signs (OD and SD) or with moderate to high parasitism (MP and HP). There was also a positive correlation between the percentage of macrophages and expression of CCL2, CCL4, and CCL5. Previous data published by our group demonstrated a decrease in absolute values of circulating monocytes as a hallmark found in the symptomatic group and in the group with the higher parasite load [6]. These data may suggest the recruitment of monocytes to other tissues during active CVL, where they might play an important role in immunological connections throughout antigen presentation and parasite clearance. However, the presence of macrophages in the skin infiltrates does not guarantee their ongoing function since histological analysis of skin during CVL described in this and other studies showed an intense cell infiltrate composed of mononuclear cells in animals with high parasitism that were clinically symptomatic [19]. The finding that expression of macrophage chemoattractants was associated with parasite burden contradicts previous *in vitro* data demonstrating that these chemokines have a macrophage-activating protective effect. This would suggest that the chemokines are recruiting immature or unresponsive macrophages. Moreover, the levels of CXCL8 observed in HP animals, despite inducing macrophage recruitment, seemed to favor the persistence of the parasite in the skin compartment. In addition, high levels of macrophages in the skin of dogs with active CVL (OD and SD) and in dogs with MP and HP density were demonstrated and highlighted the inability of these cells to control parasitism.

Our study represents the first investigation on the involvement of CCL21 in CVL. We also observed increased levels of CCL21 in animals with high parasitism, independent of the positive correlation between the chemokine and cutaneous parasitism. It has been reported that CCL21 is an important chemokine involved in recruiting antigen-presenting cells (APCs) to lymphoid organs [35]. In murine infection by *L. donovani*, Ato et al. [36] demonstrated that CCL21 is important in the marginal zone of the spleen for maintaining the structure of its cellular composition and capturing blood antigens during *Leishmania* infection. Moreover, mice deficient for the gene encoding CCL21 have greater susceptibility to infection when exposed to *L. donovani* due to the loss of dendritic cell migration [37]. In this context, we hypothesize that increased skin parasitism has the potential to stimulate the expression of CCL21, resulting in the recruitment of APCs in the skin from the lymphoid organs. However, it is possible that either these cells, like macrophages, do not present a functional profile favoring a Th1-immune response that would be effective against *Leishmania* infection. Alternately, the increase of CCL21 may lead to retention of APCs in the skin and reduce their migration to the regional lymph node where antigens would be presented to T cells.

Several studies have reported the involvement of mast cells in regulating immunity against various *Leishmania* species [38]–[40]. In the present study, a decrease of this population was observed in the skin of animals presenting severe clinical forms of the disease (OD and SD group) and in all groups categorized according to parasite density (LP, MP, and HP) when compared with the control group. This finding could be related to this cell type being involved in attempts to contain the intense skin parasite density, as described in several studies that evaluated a murine model [41]–[43]. Calabrese et al. [21] described an intense inflammatory skin reaction formed mainly by mast cells, indicating that these cells might exert a role in innate immunity against *L. infantum* infection. Our data regarding mast cells conflict with this possibility, however. The discrepancy might be explained by *L. infantum* infection causing a diverse range of clinical and histopathological manifestations. Variations in host resistance may help to explain the variations found in the skin parasite load in dogs. Moreover, when dogs from different regions are compared, additional factors must be considered, such as variations in weather conditions (e.g., *Leishmania* infection seems to occur chiefly in dry seasons).

In the present study, decreases in the eosinophil population and CCL24 expression were observed that were related to the clinical progression and skin parasite density. CCL24 is a specific agonist for CCR3, attracting and activating eosinophils in parasitic diseases [44]. Some authors have described a microbicidal capability of eosinophils against *L. donovani* and *L. major* parasites [45], [46] and suggested this cell type could play an important role in protection against *Leishmania* infection [47]. Moreover, Amusategui et al. [48] reported that eosinophil counts were higher in dogs that presented cutaneous signs, and they suggested that this finding was associated with allergenic responses. More studies are necessary to determine the role of eosinophils in the cutaneous immune response in CVL.

The participation of neutrophils in addressing infection by parasites of the genus *Leishmania* has been studied in recent years to understand the mechanisms related to the innate immune response [49]–[51]. We observed that higher CXCL8 levels existed in dogs presenting high cutaneous parasitism. This chemokine induces neutrophil chemotaxis, and the initial influx of neutrophils seems to be beneficial for the survival of *Leishmania* in the infected tissue [50]. Interestingly, it has been reported that the parasite itself produces a protein with chemoattractant properties, called *Leishmania chemotactic factor*, which promotes the migration of neutrophils to the site of infection [49], thereby boosting the phagocytosis of the parasite. Peters et al. [51] evaluated the events that occur in the skin during the initial phase of the transmission of *L. major* by sand flies and observed that a decrease in neutrophils at the infection site is associated with the inability of parasites to establish infection. This hypothesis is strongly supported by a recently published study from our group that showed a mixed cytokine profile during active CVL with predominantly higher cutaneous levels of interleukin (IL)-10 and transforming growth factor β1 apart from lower expression of IL-12. These findings might represent a key condition that allows persistence of parasite replication in the skin [17].

Herein, our data highlight the skin as an important organ in CVL and suggest that increased levels of CCL2, CCL4, CCL5, and CCL21 are associated with the immunopathogenesis of CVL. Our data also suggest that the expression of these cytokines in skin could be used as biomarkers for disease progression in dogs naturally infected by *L. infantum*. Our findings represent an advance in the knowledge of the involvement of skin inflammatory infiltrates in CVL and the systemic consequences and may contribute to developing a rational strategy for the design of new and more efficient prophylactic tools and immunological therapies against CVL.
